# Efficacy of Tranexamic Acid in the Treatment of Massive Upper Gastrointestinal Bleeding: A Randomized Clinical Trial

**DOI:** 10.7759/cureus.33503

**Published:** 2023-01-08

**Authors:** Meghdad Sedaghat, Majid Iranshahi, Maryam Mardani, Nima Mesbah

**Affiliations:** 1 Department of Adult Gastroenterology and Hepatology, Shahid Beheshti University of Medical Sciences, Tehran, IRN; 2 Student Research Committee, Shiraz University of Medical Sciences, Shiraz, IRN; 3 Department of Internal Medicine, Shahid Beheshti University of Medical Sciences, Tehran, IRN

**Keywords:** peptic ulcer bleed, gi surgery, recurrent gi bleeding, upper gastrointestinal bleeding, tranexamic acid

## Abstract

Background

Upper gastrointestinal bleeding (GIB) is an important cause of emergency ward admission. Antifibrinolytic agents including tranexamic acid (TXA) have been used for controlling GIB. However, there have been concerns regarding the safety and efficacy of TXA in patients with GIB. Thus, in this study, we aimed to determine the efficacy of TXA in the treatment of massive upper GIB.

Methodology

This double-blind randomized clinical trial was conducted among 86 consecutive patients who were referred to Imam Hossein Hospital in Tehran, Iran from 2018 to 2019 with the chief complaint of massive upper GIB. Patients were chosen to be in the TXA or placebo groups based on a 1:1 allocation using the block randomization method. The rate of rebleeding, need for blood transfusion, hospital stay, adverse effects, and mortality rate were evaluated and compared across the groups.

Results

Of the 86 patients enrolled in this study, 55.8% (n = 48) were males. The mean age of all patients was 53.1 ± 10.6 years (TXA group: 54.9 ± 11.5 years, and placebo group: 51.4 ± 9.7 years). Rebleeding was seen in 11 (25.6%) patients in the TXA group and in 20 (46.5%) patients in the control group, which was statistically significant (p = 0.043). Blood transfusion was carried out in only three (7%) patients in the TXA group compared with 14 (32.6%) patients in the placebo group (p = 0.003). Six (14%) patients experienced a hospital stay of longer than five days in the TXA group and 15 (34.9%) patients in the control group, which was statistically significant (p = 0.024). There were no significant differences in the mortality rate across both groups (p > 0.05).

Conclusions

TXA has no effect on mortality associated with severe upper GIB. However, it was associated with a lower rate of rebleeding and hospitalization time, without significant adverse effects.

## Introduction

Upper gastrointestinal bleeding (GIB) is an important cause of huge mortality and high health costs. The prevalence is the same in men and women and increases with age [1]. The mortality rate is reportedly between 3% and 14% and is mostly associated with the decompensation of underlying disease [2]. Peptic ulcer disease, followed by esophageal varices, and malignancies are considered the most common causes of GIB, which if severe, presents with hematemesis, melena, and hemodynamic instability [3,4]. Acute management of choice for patients suffering from GIB includes medical and endoscopic therapy, blood transfusion, and surgery [5,6]. Early blood transfusion is considered lifesaving among patients with rapid blood loss, and significant comorbidities, including coronary artery disease, brain injury, and any other medical condition that predispose patients to ischemic damage. However, for most GIB patients, a restrictive transfusion strategy is indicated because it is associated with better clinical outcomes [7].

Tranexamic acid (TXA), a lysine-analog antifibrinolytic agent, inhibits plasminogen activation by binding to its lysin binding site leading to the inhibition of fibrin clot degradation [8,9]. TXA has been widely used in the control of bleeding in postpartum hemorrhage and traumatic patients [10]. Furthermore, it has shown beneficial effects in patients undergoing radical retropubic prostatectomy [11]. There have been previous reports supporting the beneficial effects of TXA in the treatment of upper GIB [12]. A systematic review by Gluud et al. [13] suggested that TXA may reduce GIB mortality. In addition, they concluded that the use of TXA for GIB was not associated with an increased risk of thromboembolic events. However, there are controversial reports regarding the efficacy of TXA, and some important adverse effects have also been reported. The HALT-IT trial concluded that TXA had no effect in reducing mortality associated with GIB and is associated with an increased risk of thromboembolic events and seizure [14]. For this reason, the use of this drug requires further studies to ascertain the efficacy and safety profile of TXA. Accordingly, this study aimed to determine the efficacy of TXA in the treatment of massive upper GIB.

## Materials and methods

Study design

This double-blind, randomized clinical trial was conducted among 86 consecutive patients who were referred to Imam Hossein Hospital in Tehran, Iran from 2018 to 2019 with the chief complaint of massive upper GIB. All patients aged over 18 years, with an unstable hemodynamic state which was defined as systolic blood pressure under 90 mmHg and heart rate over 110 beats per minute, and fulfillment of informed consent were included in this study. Furthermore, patients under the age of 18 years, pregnant/breastfeeding patients, and those with contraindications for use of TXA (history of thromboembolic disorder, esophageal varicose vein bleeding, hypersensitivity to TXA, hereditary thromboembolic disorders, use of oral estrogen-containing contraceptives, heart valvular diseases, atrial fibrillation, and those requiring anticoagulant agents) were excluded from the study.

Data collection

Demographic data including age, sex, history of smoking, vital signs, and cause of GIB (as determined by upper GI endoscopy) were recorded. Furthermore, standard GIB laboratory data, including hemoglobin (Hb) level, blood group, kidney function tests (blood urea nitrogen, creatinine), prothrombin time (PT), and partial thromboplastin time (PTT), were collected. Indications for blood transfusion were considered as Hb <7 g/dL for patients without any comorbidity. The transfusion threshold was at Hb 9 g/dL for patients with life-threatening comorbidities predisposing the patient to ischemic damage.

Rebleeding was defined as hematemesis, hematochezia, significant Hb drop of more than 2 g/dL during the hospital stay, continuous melena, and bright red blood in the nasogastric tube after the treatment was completed.

Randomization and intervention

**Figure 1:**
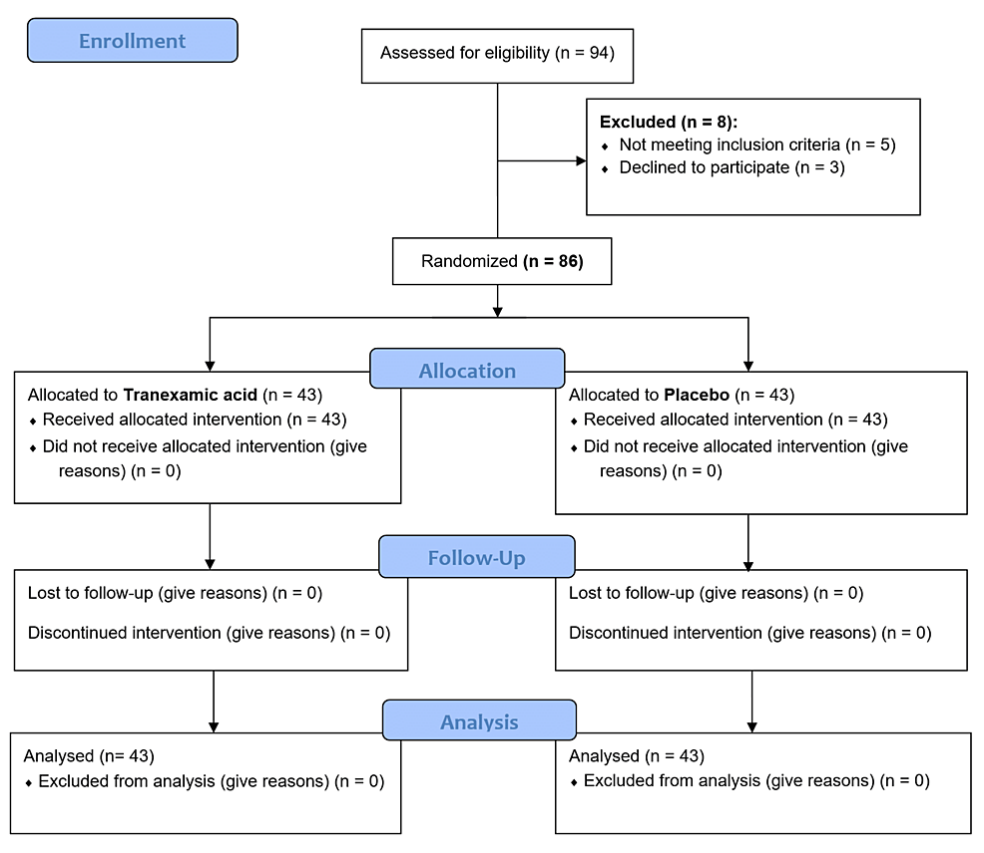
The CONSORT flow diagram of the study.

Patients were chosen to be in the TXA (500 mg vial, Abureyhan Co., Iran) or placebo groups based on a 1:1 allocation using the block randomization method and GraphPad software [15]. Both groups received standard upper GIB therapeutic approaches, including hemodynamic stability (hydration), acid suppression, upper GI endoscopy, and blood transfusion (if applicable). TXA was used intravenously for 10 minutes and then 1 g was prescribed again as a slow infusion for eight hours (Figure 1).

Statistical analysis

Data analysis was done by SPSS version 26.0 (IBM Corp., Armonk, NY, USA). Quantitative variables were reported as mean ± SD, and qualitative variables were reported as numerical (percentage) data. The Kolmogorov-Smirnov test was used for assessing the normality of data. Considering the normality of data based on the Kolmogorov-Smirnov test (p-value = 0.7), Fisher’s exact test was used for analyzing the differences between the two study groups, and a p-value under 0.05 was considered statistically significant.

Ethical consideration

This study was conducted after obtaining permission from the Medical Ethics Committee of Shahid Beheshti University of Medical Sciences with the registration number IR.SBMU.MSP.RFC.1398.238.

## Results

Of the 86 patients enrolled in this study, 55.8% (n = 48) were males. There were no significant differences between gender in both groups. The mean age of all patients was 53.1 ± 10.6 years (TXA group: 54.9 ± 11.5 years, and placebo group: 51.4 ± 9.7 years). Moreover, there were no significant differences in age between the two groups. In addition, the most common cause of upper GIB was peptic ulcer disease (83.7%, n = 72), followed by malignancy (9.3%, n = 8), Mallory-Weiss tear (2.3%, n = 2), erosive gastritis (2.3%, n = 2), esophageal ulcer (1.16%, n = 1), and angiodysplasia (1.16%, n = 1). As shown in Table 1, demographic data were similar across the groups (p > 0.05).

**Table 1:** Demographic data across the groups.

Variable		Tranexamic acid (n = 43)	Placebo (n = 43)	P-value
Age (years)	>60	17 (39.5%)	14 (32.6%)	>0.05
	<60	26 (60.5%)	29 (67.4%)	
Sex	Male	23 (53.5%)	25 (58.1%)	>0.05
	Female	20 (46.5%)	18 (41.9%)	

As shown in Table 2, rebleeding was seen in 11 (25.6%) patients in the TXA group and 20 (46.5%) patients in the control group, which was statistically significant (p = 0.043). Blood transfusion was carried out in only three (7%) patients in the TXA group. However, 14 (32.6%) patients in the placebo group needed blood transfusion (p = 0.003). Six (14%) patients experienced a hospital stay longer than five days in the TXA group and 15 (34.9%) patients in the control group, which was statistically significant (p = 0.024). There were no significant differences in the mortality rate across both groups (p > 0.05). During the trial, only one patient experienced an adverse effect, which was a mild hypersensitivity reaction presenting with a skin rash that responded to an antihistamine.

**Table 2:** Outcome measures across the groups.

Variable	Tranexamic acid (n = 43)	Placebo (n = 43)	P-value
Rebleeding	11 (25.6%)	20 (46.5%)	0.043
Blood transfusion	3 (7.0%)	14 (32.6%)	0.003
Hospital stay >5 days	6 (14.0%)	15 (34.9%)	0.024
Mortality	-	1 (2.3%)	>0.05
Adverse effects	1 (2.3%)	-	>0.05

## Discussion

Upper GIB, defined as bleeding proximal to the ligament of Treitz, is a common disease with various etiologies. According to the severity of the bleeding, initial management mainly includes airway management and intravenous hydration. Proton pump inhibitor (PPI) therapy is a non-invasive pharmacologic treatment, which reduces rebleeding, mortality, and the need for endoscopic interventions. Therapeutic endoscopy is considered the cornerstone of diagnosis and treatment of the underlying cause of GIB [16,17]. Antifibrinolytic agents including TXA have also been used for bleeding control in GIB. However, there have been concerns regarding the safety and efficacy of TXA in patients with GIB.

Our study aimed to compare the effects of TXA in the management of massive upper GIB versus placebo and to evaluate the role of TXA in bleeding control. According to this trial, it appears that TXA has beneficial effects in the treatment of upper GIB. In addition, it is associated with a significant reduction in the rate of rebleeding, blood transfusion, and hospital stay. Furthermore, the incidence of therapeutic adverse effects was the same across both groups. However, TXA did not reduce the mortality rate from GIB.

According to our study, the incidence of GIB was different among males (55.8%) and females (44.2%), which was similar to previous studies reporting a higher frequency of peptic ulcer disease in men [18]. Accordingly, TXA was associated with a lower frequency of rebleeding (25.6% in TXA groups, and 46.5% in the placebo group) compared to the standard upper GIB approach without TXA, which was statistically significant. Similar to our results, von Holstein et al. [12] concluded that the incidence of rebleeding was less in the TXA group compared to the placebo group. However, it was not statistically significant, which could be due to the limited sample size (n = 29). In addition, the HALT-IT Trial revealed no significant difference in rebleeding between both groups [14]. On the contrary, Redeen [19] reported a slight increase in the rate of rebleeding in the TXA group compared to the placebo (17.5% in the TXA group, and 13.6% in the placebo group), which was not statistically significant (p = 0.594).

Regarding the need for blood transfusion, our study showed a significant reduction in blood transfusion in the TXA group compared to the placebo group. von Holstein et al. reported similar results supporting the hypothesis that TXA reduces the need for blood product transfusion (p = 0.018) [12]. Accordingly, the HALT-IT Trial showed no significant difference in the need for transfusion in both groups [14]. Surprisingly, Redeen reported that TXA increased the need for blood transfusion (84% needed a blood transfusion in the TXA group compared to 64% in the control group, p = 0.039) [19]. Altogether, it seems highly unlikely that TXA could increase the need for blood transfusion.

TXA administration has been proven to reduce bleeding-associated death among patients with postpartum hemorrhage and trauma. The CRASH-2, a randomized placebo-controlled trial, evaluated the effects of early TXA administration on mortality and morbidity in trauma patients. They concluded that early TXA administration (beyond three hours) safely reduces mortality among traumatic patients without significant adverse effects [20]. Furthermore, previous studies have proven that TXA administration reduces mortality and bleeding in women with postpartum hemorrhage. They also suggested that TXA administration should start as soon as possible for better outcomes among patients with upper GIB [21].

Contrary to our findings, Redeen noted longer hospitalization in patients receiving TXA compared to the placebo group [19]. However, based on our trial, it seems that TXA significantly decreases the duration of hospitalization. This is mainly associated with decreased frequency of rebleeding and the need for blood transfusion.

Furthermore, TXA administration apparently had no effect on the mortality rate among patients with massive upper GIB. During this trial, only one patient passed away in the placebo group, and myocardial infarction was considered to be the cause. The HALT-IT trial suggested the same results, which was that there is no association between TXA administration and mortality reduction [14]. Previous small trials have suggested significant mortality reduction associated with TXA administration [22,23].

During our trial, only one patient treated with TXA experienced an adverse effect associated with the medication, which was a skin reaction to TXA. Compared to placebo, it seems that TXA is not associated with significant adverse effects. Previous studies support the fact that TXA is not associated with a significant increase in the risk of adverse effects [24]. In the HALT-IT trial, there was a slight increase in the risk of adverse effects, including thromboembolic events and seizures. However, it was not statistically significant [14]. It is notable to mention that a longer duration of TXA administration might increase the risk of thromboembolic events. Furthermore, a higher dosage of TXA may lower the seizure threshold and predispose patients to convulsion [25].

Notably, the main limitations of this study is the lack of a larger sample size and a short follow-up time. Therefore, we suggest future investigations to evaluate the mid-to-long-term patient outcomes with a more considerable study population. Nevertheless, this study is one of the few prospective clinical trials to assess the safety and efficacy of TXA for the management of massive upper GIB.

## Conclusions

In summary, we noted that TXA has no effect on mortality associated with severe upper GIB. However, it was associated with a lower rate of rebleeding and hospitalization time, without significant adverse effects. Thus, we encourage physicians to expand the use of TXA in the treatment of GIB outside the context of current standard treatment. Future studies with larger sample populations are required to ascertain other aspects of TXA in the treatment of GIB.
